# *Staphylococcus aureus* increases platelet reactivity in patients with infective endocarditis

**DOI:** 10.1038/s41598-022-16681-7

**Published:** 2022-07-28

**Authors:** Amin Polzin, Lisa Dannenberg, René M’Pembele, Philipp Mourikis, David Naguib, Saif Zako, Carolin Helten, Tobias Petzold, Bodo Levkau, Thomas Hohlfeld, Mareike Barth, Tobias Zeus, Stephan Sixt, Ragnar Huhn, Payam Akhyari, Artur Lichtenberg, Malte Kelm, Till Hoffmann

**Affiliations:** 1 Division of Cardiology, Pulmonology and Vascular Medicine, Heinrich Heine University Medical Center Düsseldorf, Moorenstrasse 5, 40225 Düsseldorf, Germany; 2 Cardiovascular Research Institute Düsseldorf (CARID), Düsseldorf, Germany; 3 Department of Anesthesiology, Heinrich Heine University Medical Center Düsseldorf, Düsseldorf, Germany; 4 Medizinische Klinik und Poliklinik I, Klinikum der Universität München, Ludwig-Maximilians-University Munich, Munich, Germany; 5 Institute of Molecular Medicine III, Heinrich Heine University, Düsseldorf, Germany; 6 Institute of Pharmacology and Clinical Pharmacology, Heinrich Heine University, Düsseldorf, Germany; 7 Department of Cardiovascular Surgery, Medical Faculty, Heinrich-Heine-University, Düsseldorf, Germany; 8 Transfusion Medicine and Clinical Hemostaseology, Heinrich Heine University Medical Center Düsseldorf, Düsseldorf, Germany

**Keywords:** Cardiology, Cardiovascular biology, Bacterial infection

## Abstract

Thromboembolism is frequent in infective endocarditis (IE). However, the optimal antithrombotic regimen in IE is unknown. *Staphylococcus aureus* (SA) is the leading cause of IE. First studies emphasize increased platelet reactivity by SA. In this pilot study, we hypothesized that platelet reactivity is increased in patients with SA− IE, which could be abrogated by antiplatelet medication. We conducted a prospective, observatory, single-center cohort study in 114 patients with IE, with four cohorts: (1) SA coagulase positive IE without aspirin (ASA) medication, (2) coagulase negative IE without ASA, (3) SA coagulase positive IE with ASA, (4) coagulase negative IE with ASA. Platelet function was measured by Multiplate electrode aggregometry, blood clotting by ROTEM thromboelastometry. Bleeding events were assessed according to TIMI classification. In ASA-naïve patients, aggregation with ADP was increased with coag. pos. IE (coagulase negative: 39.47 ± 4.13 AUC vs. coagulase positive: 59.46 ± 8.19 AUC, p = 0.0219). This was abrogated with ASA medication (coagulase negative: 42.4 ± 4.67 AUC vs. coagulase positive: 45.11 ± 6.063 AUC p = 0.7824). Aspirin did not increase bleeding in SA positive patients. However, in SA negative patients with aspirin, red blood cell transfusions were enhanced. SA coagulase positive IE is associated with increased platelet reactivity. This could be abrogated by aspirin without increased bleeding risk. The results of this pilot study suggest that ASA might be beneficial in SA coagulase positive IE. This needs to be confirmed in clinical trials.

## Introduction

Infective endocarditis (IE) is a severe disease, causing destruction of endocardial structures and resulting in high morbidity and mortality^1,2^. Despite all improvements in medical care and therapy, the overall mortality remains high (up to 18%) due to cardiac failure and thromboembolic complications^2^. In vitro studies suggested that increased platelet activation plays a central role in the pathophysiology of IE^3^. These studies showed that IE inducing bacteria can interact with platelets and coagulation factors^4,5^. This facilitates microbial surface binding and formation of vegetations promoting thromboembolic events^6^. Especially *Staphylococcus aureus* (SA) and its virulence factor clumping factor A (ClfA) have been shown to trigger platelet adhesion and to directly induce platelet aggregation in vitro^7,8^. Therefore, IE inducing bacteria can be divided in SA coagulase positive and coagulase negative bacteria depending on their ability to induce clotting reactions. ClfA allows SA to cover its surface with proteins and platelets from the host organism and to escape immune response^9^. As underlying mechanisms for the pro-thrombotic effects, fibrinogen and IgG dependent pathways were suggested^8,10,11^. Furthermore, SA also influences the coagulation system: it secretes soluble virulence factors, like staphylocoagulase (Coa) and von Willebrand factor-binding protein (vWbp) that directly interact with prothrombin, leading to the conversion of fibrinogen to fibrin^10,12,13^.

However, investigation on platelet function in IE-patients is rare. Therefore, we compared platelet function of patients with SA coagulase positive (SA+) and coagulase negative (SA−) IE. Furthermore, we investigate the effects of acetylsalicylic acid (ASA) as antiplatelet medication in these patients.

## Methods

### Study design, patient population

**Figure 1:**
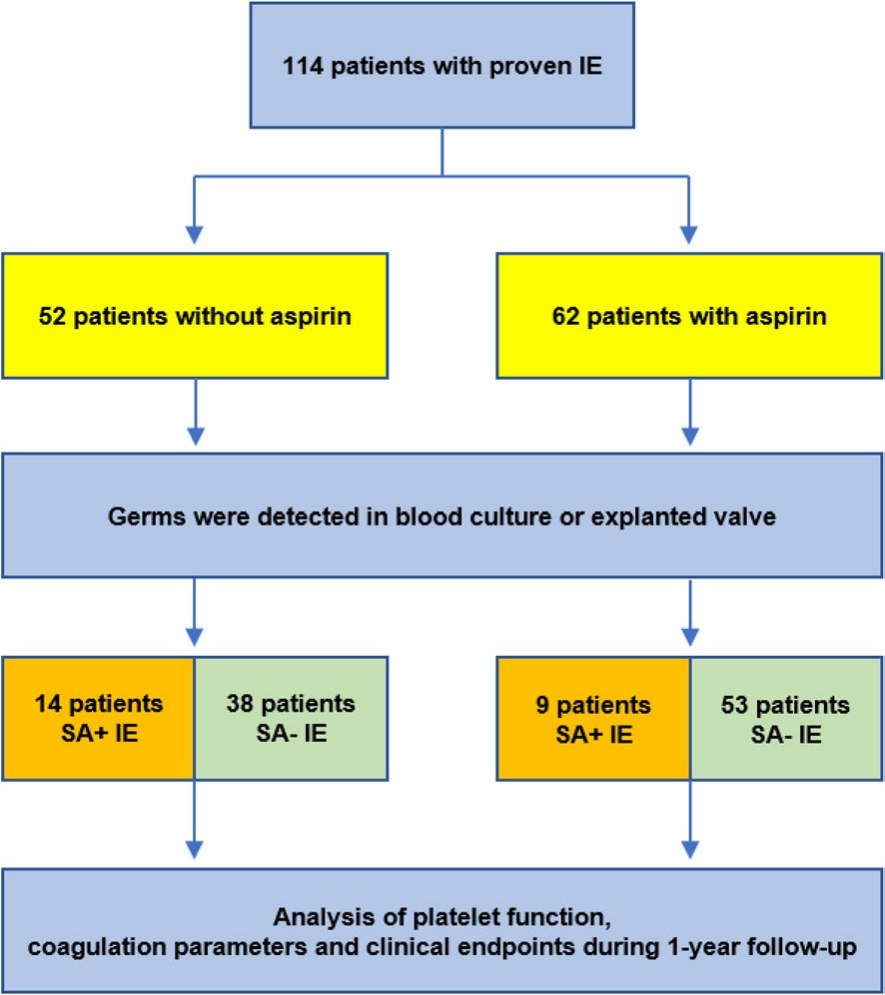
Study design as flowchart.

In this observatory, single-center cohort study, we analysed 114 patients with clinically proven IE with indication for valve surgery in the University Hospital Düsseldorf, Germany from 2013 to 2017. Informed consent was obtained from all patients. Germs were detected in blood cultures or by microbiological examination of the explanted valve respectively. Four study groups were defined depending on prior and perioperatively continued ASA medication (oral administration of 100 mg ASA *once per day*) and SA status (Fig. 1). Age under 18, coagulation disorders, haematological diseases and platelet medication others than ASA were exclusion criteria. After surgery, patients were on oral anticoagulation for 3 months, followed by indefinite single antiplatelet medication. The study conformed to the declaration of Helsinki and was approved by the Ethics Committee of the University of Düsseldorf (2018-105-RetroDEuA).

### Platelet function and coagulation parameters testing

Blood samples were collected in citrate vacutainers (1:10) immediately before valve surgery. Platelet function was measured as point of care test by multiple electrode aggregometry (MEA, Multiplate, Roche diagnostics, Germany) with adenosine diphosphate (ADP) and thrombin receptor activating peptide (TRAP) as stimulants (Roche, Germany). Aggregation was measured as area under the Curve (AUC). In addition, thromboelastometry (ROTEM sigma, Werfen, Germany) was used to evaluate specific plasmatic coagulation parameters like FIBTEM A10, EXTEMCT and INTEM CT. As standard coagulation parameters, Quick, international normalized ratio (INR), and partial thromboplastin time (PTT) were determined.

### Amount of erythrocyte transfusions

To estimate the risk of perioperative bleeding the amount of erythrocyte transfusions was evaluated for each patient. The documented number of administered erythrocyte concentrates was counted from operating theatre and intensive care unit transfusion protocols.

### Standardized bleeding assessment with thrombolysis in myocardial infarction (TIMI) bleeding criteria

Bleeding was standardized with TIMI bleeding criteria. As criteria fatal bleeding, perioperative intercranial bleeding, reoperation after closure of sternotomy to control bleeding, transfusion of more than 5 packed red blood cells within 48 h and chest tube output more than 2 L within 24 h were evaluated.

### One-year follow-up of clinical endpoints

Follow-up was conducted for clinical endpoints at 1 year after surgery. Major adverse cardiovascular and cerebrovascular events (MACCE) were assessed including stroke, death, myocardial infarction and bleeding events.

### Statistics

All statistics and analyses were conducted in IBM SPSS-Software (New York, USA) and GraphPad-Prism software (GraphPad software Inc, San Diego). Kolmogorov–Smirnov-Test and Shapiro–Wilks-Test were used to prove normal distributions. Results and descriptive statistics are presented as mean with standard deviations (mean ± SD) for continuous variables. Binary variables are presented as absolute numbers with percentage amount of the population in brackets (No. [%]). Unpaired *t* tests, One-way ANOVA and Kruskal–Wallis tests were used for comparison of continuous variables, while chi square or the Fisher’s exact test were used to analyse categorical variables. Multivariable linear regression was used to show effects of independent variables on a dependent variable. Kaplan–Meier curves with log-rank tests for trend were used to analyse results from follow-up of clinical events. P-values below 0.05 were considered significant.

### Ethics committee approval

The study conformed to the Declaration of Helsinki and was approved by the University of Düsseldorf Ethics Committee (No.: 2018–105-RetroDEuA).

## Results

### Study patients and baseline characteristics

In this study we included 114 patients with proven IE. This cohort was divided in four groups: (1) 38 patients with Coagulase negative (SA−) IE naïve to ASA, (2) 14 patients with SA+ IE naïve to ASA, (3) 53 patients with SA− IE and ASA medication and (4) 9 patients with SA+ IE and ASA medication (Fig. 1).

In patients naïve to ASA medication, overall mean age was 59.3 ± 15.3 years. 38 patients had a SA− and 14 patients a SA+ IE. Patients with SA+ IE were slightly younger (SA−: 64.1 ± 12.4 years vs. SA+: 54.5 ± 18.4 years, p = 0.034) and mostly female (SA−: 29 [76.3%] vs. SA+: 6 [42.9%], p = 0.043). Regarding other baseline parameters, comorbidities, comedication and laboratory parameters, there were no further differences between groups.

Overall mean age in the group with ASA medication was 63.16 ± 13.72 years. Within this group, 53 patients had a SA− and 9 patients had a SA+ IE. There were no significant differences between baseline parameters, comorbidities and comedication (Supplementary Tables 1, 2 and 3).

### Platelet function testing by Multiplate

**Figure 2:**
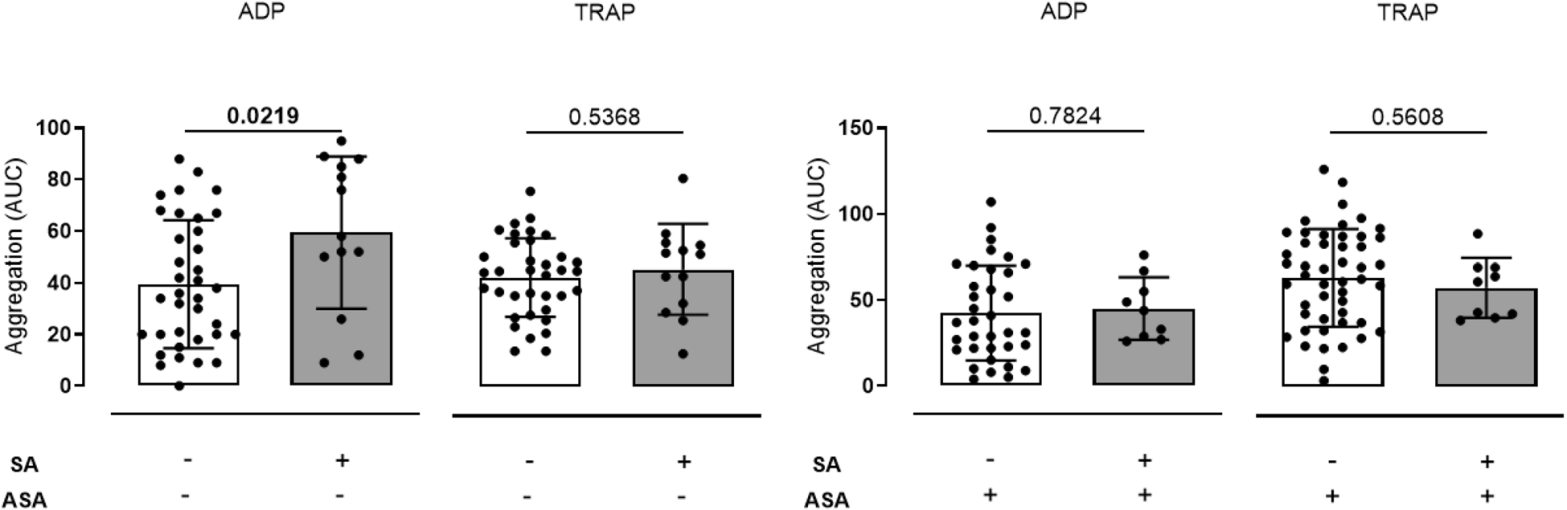
Platelet aggregation measured by Multiplate^®^ in patients with coagulase negative (n = 38/n = 53) and SA coagulase positive (n = 14/n = 9) infective endocarditis without and with antiplatelet medication. (**A**) ADP induced platelet aggregation, measured by MEA as area under the curve (AUC), was significantly higher in patients with SA+ IE in patients without antiplatelet medication while aggregation with TRAP did not differ between groups compared using two tailed *t* tests (SA−/ASA−: 39.47 ± 4.13 AUC vs. SA+/ASA−: 59.46 ± 8.19 AUC, p = 0.0219; TRAP—SA−/ASA−: 85.08 ± 5.075 AUC vs. SA+/ASA−: 91.46 ± 9.715 AUC, p = 0.5368). (**B**) ADP and TRAP induced platelet aggregation showed no differences depending on coagulase status in patients with IE and antiplatelet medication (ADP—SA−/ASA+: 42.4 ± 4.67 AUC vs. SA+/ASA+: 45.11 ± 6.063 AUC p = 0.7824; TRAP—SA−/ASA+: 84.78 ± 5.407 AUC vs. SA+/ASA+: 77.11 ± 7.725 AUC p = 0.5608).

In patients without ASA medication, mean ADP induced platelet aggregation measured by MEA, was significantly higher in those patients with SA+ IE (SA−: 39.47 ± 4.13 AUC vs. SA+: 59.46 ± 8.19 AUC, p = 0.0219). This finding was robust after adjustment for age, gender, C-reactive protein (CRP) and platelet count in a multivariate linear regression model (SA+—Standardized coefficient: 30.72, 95% CI: 14.5–46.9, p = < 0.0001; Age—Standardized coefficient: 0.48, 95% CI: 0.01–0.95, p = 0.044; Gender—Standardized coefficient: 4.79, 95% CI: − 9.67 to 19.24, p = 0.508; CRP—Standardized coefficient: 0.06, 95% CI: − 0.46 to 0.57, p = 0.824; platelet count: 0.17, 95% CI: 0.94–0.24, p = < 0.0001). Platelet reactivity did not differ for TRAP induced aggregation (TRAP—SA−: 85.08 ± 5.075 AUC vs. SA+: 91.46 ± 9.715 AUC, p = 0.5368) (Fig. 2, Table 1A, Supplementary Fig. 3).

**Table 1:** Multivariate linear regression of results on ADP induced platelet aggregation in patients with coagulase negative and SA coagulase positive IE without and with antiplatelet medication. (A) In patients without antiplatelet medication SA+ IE patients showed increased ADP induced platelet aggregation. This finding was robust after adjustments for age, gender, CRP and platelet count in multivariate analysis. (B) In patients with antiplatelet medication ADP induced platelet aggregation showed no differences. In a linear regression model, only platelet count was significantly associated with ADP induced platelet aggregation measured by Multiplate^®^. Significant values are in bold.

Variables ASA−	Unstandardized coefficients B	Standard error	Standardized coefficients beta	95% CI	p-value
**(A)**					
Coagulase positive	30.72	8.01	0.48	14.5 to 46.9	**< 0.0001**
Platelet count	0.17	0.04	0.58	0.94 to 0.24	**< 0.0001**
Age	0.48	0.23	0.26	0.01 to 0.95	**0.044**
Gender	4.79	7.16	0.08	− 9.67 to 19.24	0.508
CRP	0.06	0.26	0.03	− 0.46 to 0.57	0.824

Variables ASA+	Unstandardized coefficients B	Standard error	Standardized coefficients beta	95% CI	p-value
**(B)**					
Coagulase positive	− 4.76	8.05	− 0.70	− 20.92 to 11.40	0.557
Platelet count	0.11	0.03	0.49	0.06 to 0.17	**< 0.0001**
Age	− 0.35	0.23	− 0.19	− 0.80 to 0.11	0.129
Gender	− 3.59	5.81	− 0.07	− 15.25 to 8.07	0.539
CRP	0.84	0.46	0.23	− 0.08 to 1.75	0.072

In comparison, platelet aggregation did not differ between SA+ and SA− IE patients with ASA medication (ADP—SA−: 42.4 ± 4.67 AUC vs. SA+: 45.11 ± 6.063 AUC p = 0.7824; TRAP—SA−: 84.78 ± 5.407 AUC vs. SA+: 77.11 ± 7.725 AUC p = 0.5608). This finding remained insignificant in multivariate analysis (SA−—Standardized coefficient: − 4.76, 95% CI: − 20.92 to 11.40, p = 0.557; Age—Standardized coefficient: − 0.35, 95% CI: − 0.80 to 0.11, p = 0.129; Gender—Standardized coefficient: − 3.59, 95% CI: − 15.25 to 8.07, p = 0.539; CRP—Standardized coefficient: 0.84, 95% CI: − 0.08 to 1.75, p = 0.072; platelet count: 0.11, 95% CI: 0.06–0.17, p = < 0.0001) (Fig. 2, Table 1B).

### Platelet function testing by ROTEM

**Figure 3:**
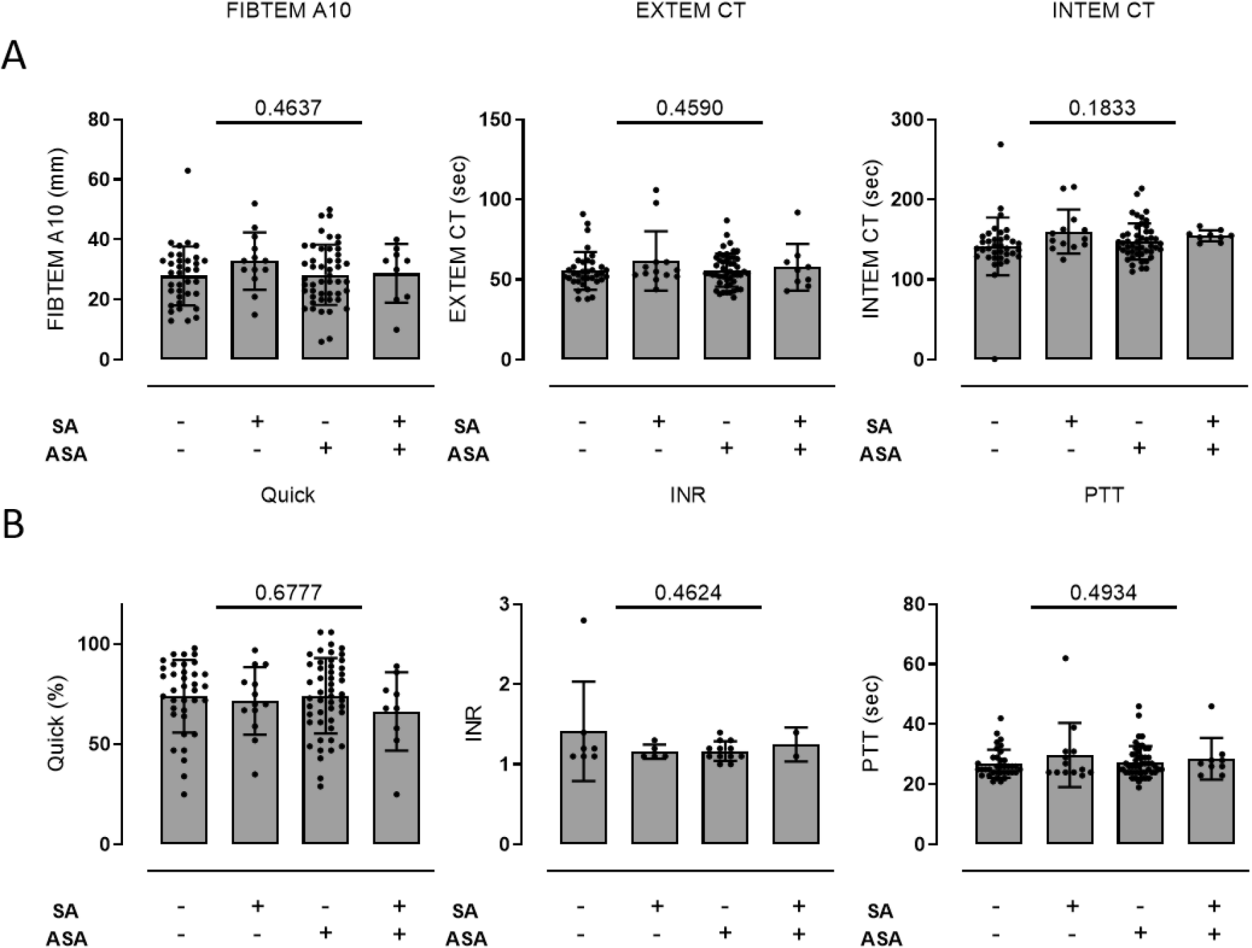
Blood clotting measured by ROTEM^®^ and coagulation parameters in patients with coagulase negative (n = 38/n = 53) and SA coagulase positive (n = 14/n = 9) infective endocarditis without and with antiplatelet medication. (**A**) Blood clotting parameters FIBTEM A10, EXTEM CT and INTEM CT measured by ROTEM^®^ showed no differences between groups (FIBTEM A10—SA−/ASA−: 27.94 ± 1.63 mm vs. SA+/ASA−: 32.85 ± 2.648 mm AUC vs. SA−/ASA+: 28.29 ± 1.45 mm vs. SA+/ASA+: 28.78 ± 3.25 mm AUC, p = 0.4637; EXTEM CT—SA−/ASA−:55.56 ± 1.945 s vs. SA+/ASA−: 61.62 ± 5.144 s vs. SA−/ASA+: 55.96 ± 1.471 s vs. SA+/ASA+: 57.78 ± 4.904 s, p = 0.4590; INTEM CT—SA−/ASA−: 141.6 ± 6.022 s vs. SA+/ASA−: 160.2 ± 7.597 s vs. SA−/ASA+: 147.8 ± 3.258 s vs. SA+/ASA+: 154.9 ± 2.318 s, p = 0.1833). (**B**) Basic coagulation parameters Quick, INR, PTT did not differ between groups (Quick—SA−/ASA−: 74.03 ± 3.021% vs. SA+/ASA−: 71.77 ± 4.684% vs. SA−/ASA+: 74.27 ± 2.687% vs. SA+/ASA+: 66.44 ± 6.539%, p = 0.6777; INR—SA−/ASA−: 1.414 ± 0.2344 vs. SA+/ASA−: 1.16 ± 0.04 vs. SA−/ASA+: 1.167 ± 0.035 vs. SA+/ASA+: 1.25 ± 0.15, p = 0.4624; PTT—SA−/ASA−: 26.83 ± 0.7802 s vs. SA+/ASA−: 29.77 ± 2.964 s vs. SA−/ASA+: 27.51 ± 0.756 s vs. SA+/ASA+: 28.56 ± 2.31 s, p = 0.4934). One-way ANOVA was used for all comparisons.

In all four groups there were no significant differences in blood clotting measured by thromboelastometry (FIBTEM A10—SA−/ASA−: 27.94 ± 1.63 mm vs. SA+/ASA−: 32.85 ± 2.648 mm AUC vs. SA−/ASA+: 28.29 ± 1.45 mm vs. SA+/ASA+: 28.78 ± 3.25 mm AUC, p = 0.4637; EXTEM CT—SA−/ASA−: 55.56 ± 1.945 s vs. SA+/ASA−: 61.62 ± 5.144 s vs. SA−/ASA+: 55.96 ± 1.471 s vs. SA+/ASA+: 57.78 ± 4.904 s, p = 0.4590; INTEM CT—SA−/ASA−: 141.6 ± 6.022 s vs. SA+/ASA−: 160.2 ± 7.597 s vs. SA−/ASA+: 147.8 ± 3.258 s vs. SA+/ASA+: 154.9 ± 2.318 s, p = 0.1833) (Fig. 3).

### Coagulation parameters

Further plasmatic coagulation parameters measured by Quick, INR, PTT and thrombin time did not differ between groups (Quick—SA−/ASA−: 74.03 ± 3.021% vs. SA+/ASA−: 71.77 ± 4.684% vs. SA−/ASA+: 74.27 ± 2.687% vs. SA+/ASA+: 66.44 ± 6.539%, p = 0.6777; INR—SA−/ASA−: 1.414 ± 0.2344 vs. SA+/ASA−: 1.16 ± 0.04 vs. SA−/ASA+: 1.167 ± 0.035 vs. SA+/ASA+: 1.25 ± 0.15, p = 0.4624; PTT—SA−/ASA−: 26.83 ± 0.7802 s vs. SA+/ASA−: 29.77 ± 2.964 s vs. SA−/ASA+: 27.51 ± 0.756 s vs. SA+/ASA+: 28.56 ± 2.31 s, p = 0.4934) (Fig. 3).

### Erythrocyte transfusions

**Figure 4:**
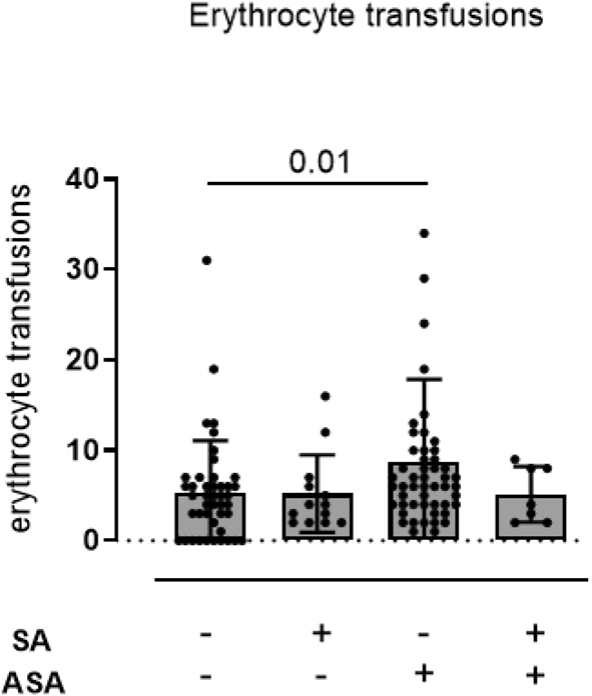
Amount of perioperative erythrocyte transfusion in patients with coagulase negative (n = 38/n = 53) and SA coagulase positive (n = 14/n = 9) infective endocarditis without and with antiplatelet medication. The amount of erythrocyte transfusion was more frequent in SA− patients with acetylsalicylic acid (ASA) medication. Red blood cell transfusions did not differ between patients without ASA and patients with SA+ endocarditis with ASA (Erythrocyte transfusions—SA−/ASA−: 5.364 ± 5.719 transfusions; SA+/ASA−: 5.231 ± 4.285 transfusions; SA−/ASA+: 8.686 ± 9.195 transfusions; SA+/ASA+: 5.143 ± 3.078 transfusions). Non-parametric Kruskal–Wallis test was used adjusted for multiple group comparison by two-stage linear step-up procedure of Benjamini, Krieger and Yekutieli.

The amount of erythrocyte transfusion did not differ between SA+ or SA− patients without ASA and SA+ patients with ASA. However, SA− patients with ASA required higher amounts of erythrocyte transfusions compared to the other groups (Erythrocyte transfusions—SA−/ASA−: 5.364 ± 5.719 transfusions; SA+/ASA−: 5.231 ± 4.285 transfusions; SA−/ASA+: 8.686 ± 9.195 transfusions; SA+/ASA+: 5.143 ± 3.078 transfusions) (Fig. 4).

### Standardized bleeding assessment with TIMI bleeding criteria

Bleeding assessment with TIMI bleeding criteria showed no significant differences between patients without ASA medication in comparison to those patients with ASA medication and SA+ or SA− IE (Supplementary Table 4).

### One-year follow-up

Patients with ASA treatment during IE before surgery had reduced mortality (Death—SA−/ASA−: 14.9% vs. SA+/ASA−: 13.3% vs. SA−/ASA+: 4% vs. SA+/ASA+: 0%; Log-rank test for trend p = 0.043). Other clinical endpoints (stroke, myocardial infarction and composite MACCE) did not differ between groups (Supplementary Fig. 1).

## Discussion

The major findings of this study were that (A) ADP stimulated platelet aggregation is higher in SA+ compared to SA− IE patient. This was independently from age, gender and CRP. (B) This was abolished in patients with ASA medication. (C) ASA did not lead to an increased rate of bleeding and need for blood transfusion.

SA has different virulence factors, which interact with coagulation factors and mediate platelet activation^8,10^. In human platelets, ClfA is considered as a main factor for activation of human platelets by SA^14^. ClfA is a staphylococcal surface protein that binds the C-terminus of human fibrinogens γ-chain^7,15^. The adherent bivalent fibrinogen molecule can bind to the platelets GPIIb/IIIa receptor and induce crosslinking^7,8^. As this crosslinking alone is insufficient to trigger platelet aggregation, a second stimulating agent is required^7^. Rapid platelet activation has been described for simultaneous binding of ClfA specific antibodies, linking ClfA to the platelets FcγRIIa-receptor, enhancing GPIIb/IIIa receptor signalling^7,16^. However, other mechanisms involving ClfA are conceivable.

ADP is a main physiological agent to induce platelet aggregation through the P2Y12- and P2Y1-receptors, located on human platelets^17^. The P2Y12-receptor is known to activate GPIIb/IIIa receptors through a phosphoinositide-3-kinase (PI3-K) pathway^17^. While ClfA bound fibrinogen is presented to the GPIIb/IIIa receptor, its activation through ADP could have synergistic effects. This mechanism could lead to amplified platelet reactivity. Additionally, we conducted in-vitro experiments which showed that ADP mediated platelet aggregation can be amplified in presence of ClfA in healthy individuals (Supplementary Fig. 2).

Besides ADP, thrombin is a strong mediator of platelet aggregation. Thrombin mainly activates human platelets through the PAR-1 receptor^18^. SA and other bacteria can induce endogenous thrombin formation through systemic inflammatory reactions^19,20^. This increase of endogenous thrombin can lead to a decrease of PAR-1 expression on platelets already at very low persistent concentrations^21^. The decrease of PAR-1 is described as time and dose dependent and could be shown in patients with systemic inflammation^21^. Ex-vivo, PAR1 down-regulation comes with a decreased responsiveness to TRAP in MEA^21^. These two mechanisms could explain our findings that platelets of patients with coagulase positive IE had an increased responsiveness to ADP dependent platelet aggregation while TRAP dependent platelet aggregation did not differ in our study.

Beside of pathologically increased platelet activation by SA, other mechanisms should be considered. There is evidence that bloodstream infections with SA can lead to a massive inflammatory response^22^. In this context CRP, as common inflammatory marker, is increased. Studies showed that elevated CRP levels can affect platelet function^23,24^. Other factors that might influence human platelet aggregation and must be considered as confounding parameters are age and gender^25^. In our study, we identified that patients with SA+ IE were slightly younger and less often male compared to SA− patients. No other differences were detected between the study groups. But even after adjustments for age, gender and CRP our findings remained robust in multivariate analysis.

We also investigated thromboelastometry and coagulation parameters in patients with SA+ IE compared to patients with SA− IE. No significant differences could be shown, although previous studies suggest that SA impairs plasma clotting times and increases PTT by secreting extracellular soluble virulence factors Staphopain A and Staphopain B^26^.

Clinical studies have shown that patients with IE caused by SA show higher morbidity and mortality^2,27^. This primarily results from association of SA caused IE with higher rates of thromboembolic events leading to major neurological events and poor outcomes^28,29^. In this study we found that ASA treatment prior to surgery was associated with reduced 1 year mortality. However, this must be interpreted with caution. All patients had oral anticoagulation for 3 months after surgery. Additionally, the study size was not powered for multivariate analysis regarding clinical endpoints. Therefore, this has to be tested in randomized trials. However, it is well known that thromboembolic events have impact on prognosis of patients with SA+ IE, therefore antithrombotic medication might play a significant role to improve outcome^29^.

Previous research on IE could show that continuous ASA medication reduces the number of thromboembolic events and reduces vegetation size, confirming the importance of platelet reactivity^30–33^. However, data on this topic was controversial because some studies identified higher bleeding rates in IE patients on ASA therapy^34,35^. However, data did not show that ASA prevented thromboembolic events, which goes in line with our findings. Hence, the European Guidelines for treatment of IE do not yet recommend antiplatelet medication^36^. These studies did not investigate SA+ versus SA− IE patients. However, based on our results, patients with SA+ IE could particularly benefit of ASA medication. This is even underlined by the fact that ASA medication seemed to be safe in our study in SA+ patients. However, SA− patients with ASA had enhanced perioperative bleeding according to RBC transfusions. Postoperative bleeding, leading to reoperation also occurred more often in these patients. Although these findings were barely significant and case number was limited, it might underline the procoagulant effects of SA on the one hand and on the other hand emphasizes the safety of ASA medication in these patients.

### Study limitations

Our study has several limitations. First it was an observatory, single-center cohort study so external validity might be limited. There is a selection bias because only patients prior to surgical treatment were included, which might have led to a lower amount of SA+ patients. Indication for surgical treatment were not recorded. This could have influenced the results as valvular dysfunctions can impact platelet function tests. Furthermore, this study used point of care tests for evaluation of coagulation and platelet function that were available in our clinical setting. Therefore, we decided to carry out platelet function tests from citrated blood to meet the standards of our local laboratory, although hirudinized blood might be the standard for multiple electrode aggregometry. This might have influenced our findings. Also, it might have been interesting to further evaluate P-selectin levels and GPIIbIIIa activation which might have been more precise to evaluate platelet activation. However, these methods were not available in our daily clinical routine. Additionally, most of the patients did not reach the reference range of ADP induced platelet function test. Therefore, it is difficult to say if SA does increase platelet aggregation in general. However, we found that SA+ patients had higher platelet aggregation as compared to control. The trial was not randomized or controlled. Therefore, other potential confounders that were not included in the multivariable analysis might have biased the results.

## Conclusion

This study showed that platelet aggregation is higher in ASA medication naïve patients with SA+ IE compared to patients with SA− E. This finding was abolished in patients with ASA medication, whereas bleeding was not enhanced in these patients. This leads to the hypothesis that ASA might be beneficial in SA+ endocarditis. Randomized clinical trials are needed to test this hypothesis.

## Supplementary Information

Supplementary Information.

## Data Availability

The datasets used and/or analysed during the current study available from the corresponding author on reasonable request.

## Abbreviations

ADP: Adenosine diphosphate
ASA: Acetylsalicylic acid
AUC: Area under the curve
ClfA: Clumping factor A
CRP: C-reactive protein
IE: Infective endocarditis
INR: International normalized ratio
LTA: Light transmission aggregometry
MACCE: Major adverse cardiovascular and cerebrovascular events
MEA: Multiple electrode aggregometry
PRP: Platelet rich plasma
PTT: Partial thromboplastin time
ROTEM: Rotational thromboelastometry
SA: *Staphylococcus aureus*
SA+: *Staphylococcus aureus* Coagulase positive
SA−: *Staphylococcus aureus* Coagulase negative
TIMI: Thrombolysis in myocardial infarction
TRAP: Thrombin receptor activating peptide
