# Supplementary material for: Staphylococcus aureus increases platelet reactivity in patients with infective endocarditis
Source: Sci Rep. 2022 Jul 28;12:12933. doi: 10.1038/s41598-022-16681-7 (PMC9334290; doi:10.1038/s41598-022-16681-7)
Supplement: Supplementary file 1 — Supplementary Information. [file 41598_2022_16681_MOESM1_ESM.docx]

**Supplementary material:**

**Supplementary figure 1:**

**
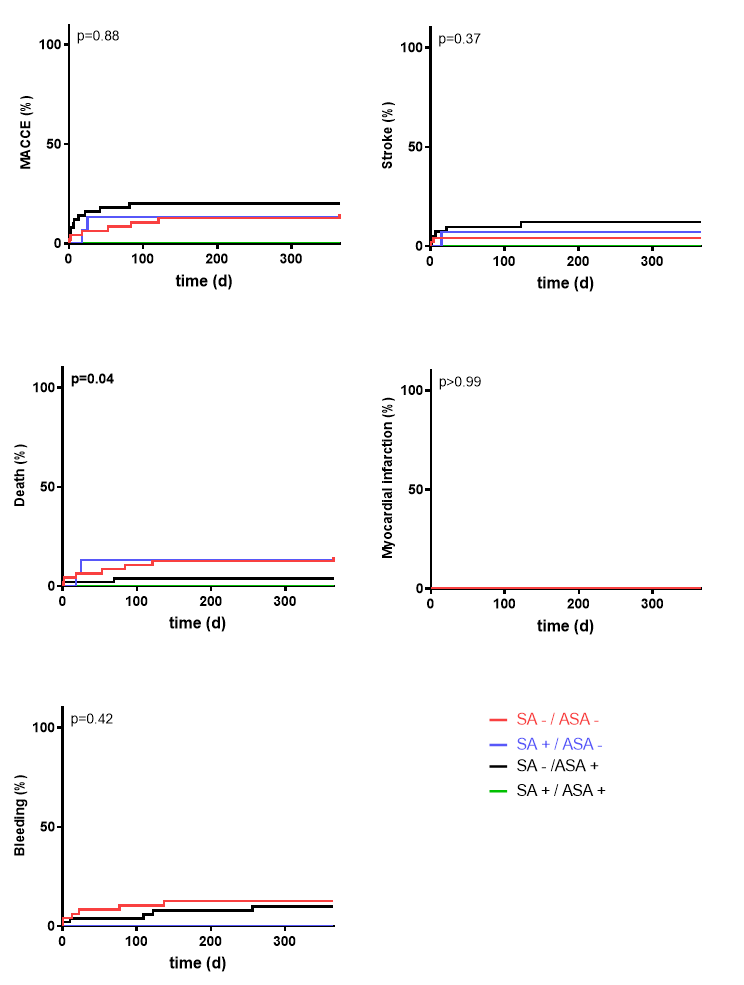
**

**Supplementary figure 1: 1-year follow-up of clinical endpoints depending on Staphylococcus aureus and ASA medication status.** The figure shows evaluation of clinical endpoints (MACCE, stroke, death, myocardial infarction and overall bleeding) comparing patients with and without staphylococcus aureus (SA) positive (+) or negative (-) infective endocarditis with (+) and without (-) ASA medication.

**Supplementary figure 2:**


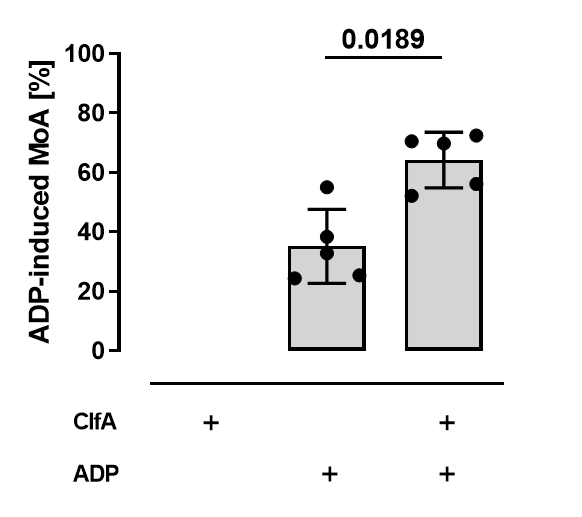


**Supplementary figure 2: Amplification of ADP induced aggregation in presence of Clumping factor A in healthy individuals.**  For light transmission aggregometry (LTA) blood samples from healthy individuals were collected in citrate vacutainers (dilution 1:10). Platelet function was measured in platelet rich plasma. Platelet rich plasma and platelet poor plasma were generated by 10 minutes of whole blood centrifugation at 270G and 1200G respectively. Adenosine diphosphate (ADP) was used as agonists in a final concentration of 10µM. ClfA was used in a final concentration of 2.8µg/ml. The figure shows that in-vitro ADP induced aggregation can be amplified in presence of Clumping factor A (ClfA) in plasma of healthy individuals (N=5) measured by light transmission aggregometry. ClfA alone showed no platelet aggregation (ADP – 35.21±12.44 vs. ADP+ClfA 64.20±9.35). Two-tailed paired t-test was used for comparison.

**Supplementary figure 3:**


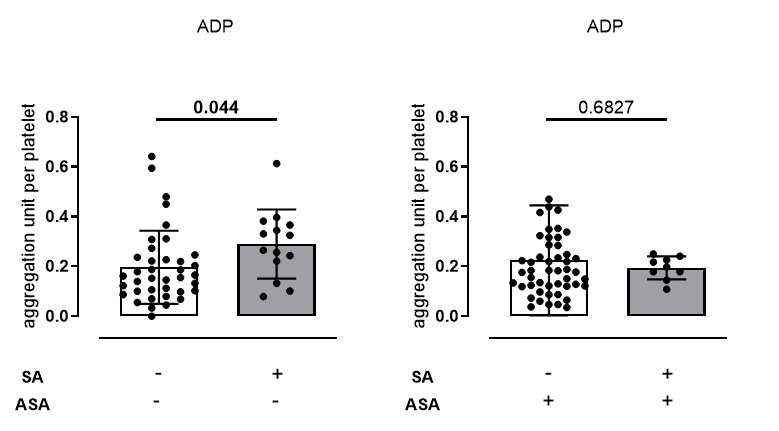


**Supplementary figure 3: ADP induced platelet aggregation normalized for platelet count in patients with infective endocarditis with or without platelet medication.** The figure shows that ADP induced platelet aggregation remained increased in patients with SA+ IE without antiplatelet medication as compared to patients with SA- IE without antiplatelet medication, when adjusted for platelet count (SA+ ASA- 0.29±0.14 U/platelet vs. SA- ASA- 0.19±0.15 U/platelet; p=0.044). Aggregation did not differ between SA- and SA+ patients with antiplatelet medication (SA+ ASA- 0.19±0.05 U/platelet vs. SA- ASA- 0.22±0.22 U/platelet; p=0.683). Two-tailed unpaired t-tests were used for comparison.

**Supplementary table 1: Baseline characteristics and comorbidities (patients without antiplatelet medication)**

|  | **SA-/ASA-**  **Group (N=38)** | **SA+/ASA-**  **Group (N=14)** | **SA-/ASA+**  **Group (N=53)** | **SA+/ASA+**  **Group (N=9)** |
| --- | --- | --- | --- | --- |
| **Characteristics** |  |  |  |  |
| Age (years) –mean ± S.D. | 64.13 ± 12.4 | 54.5 ± 18.1 | 64.15 ± 12.57 | 57.33 ± 19.06 |
| Male gender – no. (%) | 29 (76.3%) | 6 (42.9%) | 33 (62.3%) | 5 (55.6%) |
| Body Mass Index (kg/m²) – mean ± S.D. | 25.6 ± 4.4 | 26 ± 6.4 | 26.8 ± 5.73 | 27.09 ± 5.95 |
| **Comorbidities – no. (%)** |  |  |  |  |
| Hypertension | 20 (52.6%) | 4 (28.6%) | 42 (79.2%) | 7 (77.8%) |
| Obesity* | 7 (18.4%) | 3 (21.4%) | 11 (20.8%) | 2 (22.2%) |
| Diabetes | 7 (18.4%) | 2 (14.3%) | 16 (30.2%) | 1 (11.1%) |
| Hypercholesterolemia | 9 (23.6%) | 2 (14.3%) | 24 (45.3%) | 4 (44.4%) |
| Reduced ejection fraction** | 5 (13.2%) | 1 (7.1%) | 3 (5.7%) | 2 (22.2%) |
| Atrial fibrillation | 16 (42.1%) | 2 (14.3%) | 16 (30.2%) | 3 (33.3%) |
| COPD | 2 (5.3%) | 1 (7.1%) | 4 (7.5%) | 1 (11.1%) |
| Prior MI | 1 (2.6%) | 0 (0%) | 9 (17.0%) | 1 (11.1%) |
| Prior PCI | 5 (13.2%) | 1 (7.1%) | 6 (11.3%) | 1 (11.1%) |
| Prior Stroke | 7 (18.4%) | 1 (7.1%) | 16 (30.2%) | 1 (11.1%) |
| Prior CABG | 1 (2.6%) | 0 (0%) | 6 (11.3%) | 0 (0%) |
|  |  |  |  |  |

*Obesity was defined as BMI >30 kg/m²

**reduced ejection fraction was defined as systolic left ventricular ejection fraction < 45 %

COPD= Chronic obstructive pulmonary disease, MI =Myocardial infarction, PCI =Percutaneous coronary intervention, CABG=Coronary artery bypass graft

**Supplementary table 2: Comedication (patients without antiplatelet medication)**

|  | **SA-/ASA-**  **Group (N=38)** | **SA+/ASA-**  **Group (N=14)** | **SA-/ASA+**  **Group (N=53)** |  | **SA+/ASA+**  **Group (N=9)** |  |
| --- | --- | --- | --- | --- | --- | --- |
| **Comedication – no. (%)** |  |  |  |  |  |  |
| ASA | 0 (0%) | 0 (0%) | 53 (100%) |  | 9 (100%) |  |
| P2Y12 inhibition | 0 (0%) | 0 (0%) | 0 (0%) |  | 0 (0%) |  |
| Anticoagulation | 21 (55.3%) | 4 (28.6%) | 16 (30.2%) |  | 3 (33.3%) |  |
| Beta blockers | 22 (57.9%) | 5 (35.7%) | 32 (61.5%) |  | 6 (66.7%) |  |
| ACE inhibitors | 8 (21.1%) | 3 (21.4%) | 11 (21.1%) |  | 3 (33.3%) |  |
| ATII receptor antagonists | 7 (18.4%) | 0 (0%) | 3 (5.8%) |  | 1 (11,1%) |  |
| Ca Antagonists | 4 (10.5%) | 2 (14.3%) | 9 (17.3%) |  | 3 (33.3%) |  |
| Diuretics | 13 (34.2%) | 4 (28.6%) | 31 (59.6%) |  | 5 (55.6%) |  |
| Aldosterone antagonists | 3 (7.9%) | 1 (7.1%) | 6 (11.5%) |  | 2 (22.2%) |  |
| Statins | 13 (34.2%) | 3 (21.4%) | 23 (44.2%) |  | 4 (44.4%) |  |
| Oral antidiabetics | 3 (7.9%) | 2 (14.3%) | 9 (17.3%) |  | 0 (0%) |  |
| Insulin | 2 (5.3%) | 1 (7.1%) | 3 (5.8%) |  | 1 (11.1%) |  |
| Proton pump inhibitors | 23 (60.5%) | 6 (42.9%) | 43 (82.7%) |  | 8 (88.9%) |  |
| Dipyrone | 1 (2.6%) | 1 (7.1%) | 0 (0%) |  | 0 (0%) |  |
| Ibuprofen | 0 (0%) | 0 (0%) | 0 (0%) |  | 0 (0%) |  |
|  |  |  |  |  |  |  |

ACE=Angiotensin converting enzyme, AT=Angiotensin, Ca=Calcium

**Supplementary table 3: Laboratory parameters (patients without antiplatelet medication)**

|  | **SA-/ASA-**  **Group (N=38)** | **SA+/ASA-**  **Group (N=14)** | **SA-/ASA+**  **Group (N=53)** | **SA+/ASA+**  **Group (N=9)** |
| --- | --- | --- | --- | --- |
| **Laboratory parameters (mean ± S.D.)** |  |  |  |  |
| GRF (ml/min) | 66.95 ± 28.73 | 74.86 ± 40.06 | 59.94 ± 29.02 | 66.33 ± 35.04 |
| Creatinine (mg/dl) | 1.42 ± 1.28 | 1.33 ± 1 | 1.55 ± 1.22 | 1.30 ± 0.73 |
| Urea (mg/dl) | 40.89 ± 26.82 | 55.86 ± 44.91 | 48.45 ± 36.92 | 55.75 ± 48.27 |
| Cholesterol (mg/dl) | 135.4 ± 39.04 | 114.5 ± 27.58 | 145.5 ± 59.68 | 141.6 ± 39.82 |
| TG (mg/dl) | 115.71 ± 48.58 | 154 ± 18.03 | 138.44 ± 70.78 | 84.5 ± 17.99 |
| Fibrinogen (mg/dl) | 350.26 ± 166.9 | 433.33 ± 165.4 | 385.8 ± 147.01 | 636.0 ± 110.31 |
| Creatine kinase (CK) (U/l) | 426.9 ± 618.3 | 285 ± 347.9 | 115.24 ± 279.89 | 46.57 ± 31.63 |
| CK-MB (U/l) | 105.6 ± 143.6 | 46.6 ± 31.2 | 22.75 ± 11.52 | - |
| High sensitive troponin (ng/l) | 855 ± 1475.54 | 204.5 ± 239.02 | 93.42 ± 123.48 | 143.57 ± 330.86 |
| C-reactive protein (mg/dl) | 7.81 ± 13.8 | 10.48 ± 9.42 | 7.06 ± 5.80 | 10.32 ± 9.99 |
| Leukocytes x1000/µl | 11.85 ± 5.71 | 13.25 ± 6.04 | 10.16 ± 4.83 | 10.72 ± 8.87 |
| Thrombocytes x1000/µl | 209.97 ± 94.51 | 180.69 ± 102.18 | 233.56 ± 105.16 | 246.33 ± 113.10 |
| Hb (g/dl) | 9.8 ± 1.56 | 10.2 ± 1.76 | 10.53 ± 1.74 | 10.65 ± 1.99 |
| Hct (%) | 29.47 ± 4.52 | 30.51 ± 4.33 | 32.44 ± 4.73 | 33.83 ± 5.19 |
| HbA1c (%) | 17.7 ± 19.38 | 35.93 ± 29.15 | 5.64 ± 0.74 | 5.97 ± 2.17 |
|  |  |  |  |  |

GFR=Glomerular filtration rate, HDL=High density lipoprotein, LDL=Low density lipoprotein, TG=Triglycerides, CK-MB=Muscle Brain type creatine kinase, Hb=hemoglobin, Hct=Hematocrit, HbA1c=Glycated hemoglobin

**Supplementary table 4: Standardized perioperative bleeding assessment with TIMI bleeding criteria:**

|  |  | **Staphylococcus aureus positive** | | | **Staphylococcus aureus negative** | | |  |
| --- | --- | --- | --- | --- | --- | --- | --- | --- |
|  |  | **ASA+ (n=9)** | **ASA- (n=14)** | **P Value^*^** | **ASA+ (n=53)** | **ASA- (n=38)** | **P Value^*^** |  |
|  |  |  |  |  |  |  |  |  |
| **Fatal bleeding** |  | 0 (0%) | 0 (0%) | - | 1 (1.9%) | 1 (2.6%) | 0.99 |  |
| **Perioperative intracranial bleeding** |  | 0 (0%) | 0 (0%) | - | 1 (1.9%) | 2 (5.2%) | 0.569 |  |
| **Reoperation after closure of sternotomy to control bleeding** |  | 0 (0%) | 1 (7.1%) | >0.999 | 6 (11.3%) | 3 (7.9%) | 0.73 |  |
| **Transfusion of ≥5PRBCs within 48h** |  | 3 (33.3%) | 5 (35.7%) | 0.99 | 34 (64.2%) | 23 (60.5%) | 0.208 |  |
| **Chest tube output >2l within 24 hours** | |  | 0 (0%) | 0 (0%) | - | 1 (1.9%) | 2 (5.2%) | 0.569 |
